# Coronary artery bypass grafting in patients with severe left ventricular dysfunction. The role of myocardial viability

**DOI:** 10.1186/1749-8090-8-S1-P125

**Published:** 2013-09-11

**Authors:** O Zelenchuk, B Todurov, V Shevchenko, O Shnyrkova, M Rotar, O Loskutov, I Kuzmich

**Affiliations:** 1 Kiev City Heart Center, Kiev, Ukraine

## Background

The aim of this study was to identify preoperative factors predictive of a favorable outcome, and assess functional improvement after CABG in patients with ischemic cardiomyopathy and evaluate benefit of myocardial viability assessment for stratifying risk and selecting patients with low ejection fraction for coronary artery bypass grafting.

## Methods

Between 2008 and 2012, 118 patients who underwent CABG using cardiopulmonary bypass and had a preoperative LVEF less than or equal to 35% were included. LVEF was determined by echocardiography and ventriculography during left heart catheterization. Indication for surgery was predominance of tissue viability. Functional improvement was evaluated through echocardiography and scintigraphy.

## Results

Mean age of the patients was 68 ± 9 years, and 71.1% of patients were men. Mean LVEF was 27.6 ± 4.6% (ranged from 9% to 35%); mean end-diastolic volume was 205 ± 53 ml (ranged 108 ml to 348 ml). Averages of 3.2 coronary bypass grafts per patient were performed. Operative mortality was 2.5% (3 patients). After the surgery showed improvement of myocardial contractility. LVEF in the first day after surgery was 29.6 ± 4.2%, and by the seventh - has grown to 43.5 ± 4.7%, p<0,001. Left ventricular end-diastolic volume decreased to 170 ± 46 ml, p<0,001. According to the myocardial perfusion scintigraphy have shown a declining areas of hypoperfusion at 18.9 ± 7.4%, p<0,001.

## Conclusions

Myocardial revascularization for ischemic cardiomyopathy is associated with good functional relief from the symptoms of angina initially and, to a lesser extent, heart failure. Revascularization may have the advantage of preserving the remaining left ventricular function. The obtained results of surgical treatment of patients with cardiomyopathy show the effectiveness of CABG surgery, which allows us to recommend this technique as an operation of choice for this group of patients.

